# Educational impact and cost efficiency of AI-enhanced videos in pediatric surgery training: a quasi-experimental study

**DOI:** 10.1038/s41598-026-39961-y

**Published:** 2026-03-12

**Authors:** Ahmed Abdelmohsen, Nesma Elshehawy, Mohammed Abdel Razek, Refaat Badawy, Shady Sharaf, Ola Abdelmohsen, Hadir Abdelmohsen, Mohamed Abdelmohsen

**Affiliations:** 1 Department of Pediatric Surgery, Faculty of Medicine, Al-Azhar University, Cairo, Egypt; 2 Consultant of Clinical Pathology, Ministry of Health and Population, Cairo, Egypt; 3 Department of Mathematics Faculty of science, Al-Azhar University, Cairo, Egypt; 4 Department of Pediatrics, Faculty of Medicine, Al-Azhar University, Cairo, Egypt; 5 Independent Researcher in Accounting, Faculty of Commerce, Al-Azhar University, Cairo, Egypt; 6 Independent Researcher in Advanced Statistics, MSc in Statistical Sciences , Faculty of Graduate Studies for Statistical Research, Cairo University, Cairo, Egypt; 7 Department of Economics, Faculty of Commerce, Suez Canal University, Ismailia, Egypt

**Keywords:** Pediatric surgery education, Artificial intelligence, Video-based learning, Medical education technology, Educational cost analysis, Generative AI, Multimedia learning, Surgical training, Health care, Medical research

## Abstract

Pediatric surgery training is challenged by declining clinical exposure and limited access to high-quality educational resources. Advances in generative artificial intelligence (AI) allow enhancement of educational videos without professional studio infrastructure, but evidence supporting their educational value remains limited. To evaluate the educational impact and cost efficiency of locally deployed AI-enhanced lecture videos compared with standard faculty-recorded videos in pediatric surgery training. A quasi-experimental, non-randomized, post-test-only study conducted at Al-Azhar University, Cairo, Egypt between December 2024 and September 2025. Medical students and pediatric surgery residents viewed standardized lecture videos on five core pediatric surgery topics. Identical scripts and narration were used in both groups; the intervention differed only by AI-based background enhancement using open-source, locally deployed tools. Primary outcomes were perceived educational clarity (five-point Likert scale) and knowledge acquisition assessed by a 40-item multiple-choice examination. Secondary outcomes included visual comfort, perceived professionalism, and willingness to recommend the format. Between-group comparisons and effect sizes were calculated. A cost analysis compared AI-enhanced production with traditional studio-based methods. A total of 240 participants were included (120 per group). AI-enhanced videos were associated with higher educational clarity scores (mean 4.52 vs. 3.88; p < 0.001) and higher knowledge scores (76.8% vs. 69.5%; p < 0.001). Secondary outcomes consistently favored AI-enhanced videos, including visual comfort and perceived professionalism. Locally deployed AI production reduced per-video costs by 72–94% compared with traditional studio production. Locally deployed AI-enhanced videos improved short-term educational outcomes and substantially reduced production costs while preserving presenter authenticity. This approach represents a practical, scalable strategy for enhancing pediatric surgery education, particularly in resource-limited settings. Further studies are needed to assess long-term retention and clinical impact.

**Supplementary Information:**

The online version contains supplementary material available at 10.1038/s41598-026-39961-y.

## Introduction

 Pediatric surgery education faces significant challenges in contemporary medical training. General surgery residents complete only 33.8 designated pediatric cases (3.7% of total operative volume)^1^, with the ACGME requiring only 20 cases for graduation^2^. Nearly 60% of pediatric surgery experience concentrates in inguinal and umbilical hernia repairs^3^. Graduating residents demonstrate variable competence in pediatric procedures, with 97.3–97.6% competent for umbilical hernia repairs but only 79.5% competent for inguinal hernia repair in infants under 6 months^4^. Pediatric surgery case volume for general surgery residents has decreased by approximately 30.7% over the past decade^5^.

The COVID-19 pandemic exacerbated these challenges. Pediatric surgery residents experienced 46–57% reduction in operative procedures, with 70% reporting decreased surgical confidence^6^. These disruptions highlighted the need for innovative educational approaches that can supplement limited operative exposure.

Contemporary medical education increasingly embraces Education 4.0 principles emphasizing student-centered, technology-enhanced learning. Video-based education has emerged as a valuable tool in surgical training. Meta-analyses demonstrate equivalent effectiveness to traditional teaching^7^. Studies demonstrate significant normalized learning gains from video-based education, with interactive videos achieving 49–70% gains depending on format^8^. Video-based learning shows large effects on knowledge acquisition in dentistry (Cohen’s d = 2.18) and moderate effects in medicine (Cohen’s d = 0.67)^9^. These findings support video as an effective educational modality, though quality and production values vary considerably.

The Cognitive Theory of Multimedia Learning suggests that learners process visual and verbal information through limited cognitive channels, and that well-designed multimedia presentations can reduce extraneous cognitive load and support comprehension^10^. Enhanced visual presentation may improve engagement and learning outcomes without altering content delivery.

However, video production in non-studio settings requires optimization of set, lighting, sound, and video equipment, with few formal resources existing to guide educators for high-quality video production^11^. High-quality video production typically requires professional studios with substantial costs, including studio rental, equipment, and videographer services. Green screen chroma keying presents substantial technical challenges: uneven lighting causing shadows and hot spots, color spill requiring extensive correction, shadows interfering with keying, six-foot separation requirements, wardrobe restrictions, even diffused lighting across entire screen, and extensive post-production color matching. Green screen infrastructure requires dedicated studio space, professional costly lighting equipment, and technical expertise, with periodic replacement needed to avoid student disengagement.

Artificial intelligence (AI) offers new possibilities for educational video enhancement. Generative AI can transform educational content into multimodal formats, potentially improving comprehension^12^. However, current AI applications in surgical education face limitations including bias in algorithms, concerns about transparency, and risks of over-reliance^13^.

Concerns also exist regarding the use of generative AI in medical education, particularly related to anatomical accuracy and visual realism. Fully synthetic AI-generated presenters may introduce distortions or implausible features, underscoring the importance of controlled and limited AI application. Current generative AI models can produce “hallucinations,” and text-to-video models may generate anatomically incorrect motion which is defined as convincing yet false outputs^14^.

Online AI video enhancement platforms often produce color mismatches and inconsistent results. Local deployment with selective masking offers superior control. No published studies directly compare locally deployed AI-enhanced videos to standard faculty-recorded videos for pediatric surgical teaching. This represents a critical research opportunity, particularly given declining exposure and need for accessible, cost-effective solutions in resource-limited settings.

Our objective was to evaluate the impact of locally deployed AI-enhanced video presentation on educational clarity and knowledge acquisition in pediatric surgical teaching compared with standard recordings, and to assess learner satisfaction and production costs. We hypothesized that AI-enhanced videos would improve educational outcomes while substantially reducing costs.

## Methods

### Study design and participants

**Fig. 1:**
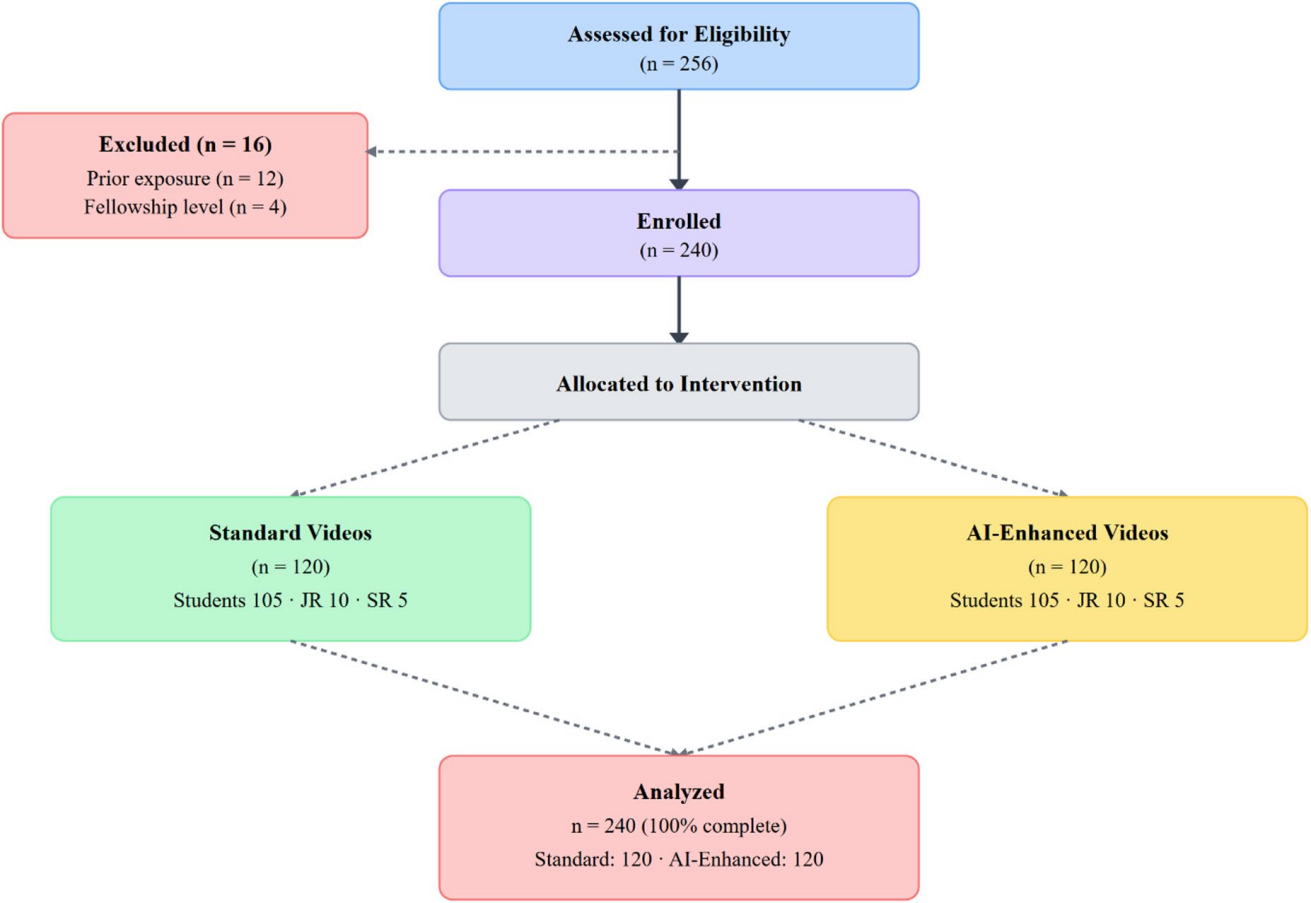
Flow diagram of the study.

This quasi-experimental, non-randomized, educational evaluation used a post-test-only design conducted at Faculty of Medicine, Al-Azhar University, Cairo, Egypt. (December 2024–September 2025). Participation was voluntary, and informed consent was obtained electronically or verbally before participation in the study. All study procedures were conducted in accordance with the ethical standards of the institutional research committee and with the principles of the Declaration of Helsinki and its later amendments. Figure 1 presents the study flow diagram.

Participation was voluntary. Eligible participants included clinical medical students (years 3–5) and pediatric surgery residents (postgraduate years ≥ 1). Exclusion criteria were prior exposure to study videos or pediatric surgery fellowship enrollment. Participants were allocated non-randomly based on scheduled teaching sessions, which were predetermined by the academic timetable and independent of individual learner characteristics. Sessions were separated by ≥ 48 h to reduce contamination between groups.

Participants were informed during the consent process that the study was evaluating different educational video formats for pediatric surgery teaching, but they were not told which format they would view or the specific hypothesis being tested. After viewing their assigned video, participants could potentially recognize differences in visual presentation quality between formats. This lack of complete blinding may have influenced subjective ratings, particularly for perceived professionalism and willingness to recommend.

## Educational content

Five topics were selected based on clinical relevance: functional constipation, undescended testes, bowel management programs, hypospadias, and ureteropelvic junction obstruction. Standardized 6–10 min lecture scripts were developed by board-certified pediatric surgeons. Identical scripts, narration, and illustrations were used in both groups to isolate visual enhancement effects.

## Video production

Recording protocol Videos were recorded using smartphone camera (4 K) on tripod with faculty against plain walls. No specialized studio or green screen was required. Audio was captured using low-cost lavalier microphone ($20–50), with professional quality achieved through Adobe Podcast’s free web-based enhancement tool.

Standard video production Standard videos underwent basic editing including trimming silences, removing pauses, color adjustment, and overlay of expert-curated illustrations using DaVinci Resolve (1–2 h per video, common to both conditions).

**Fig. 2:**
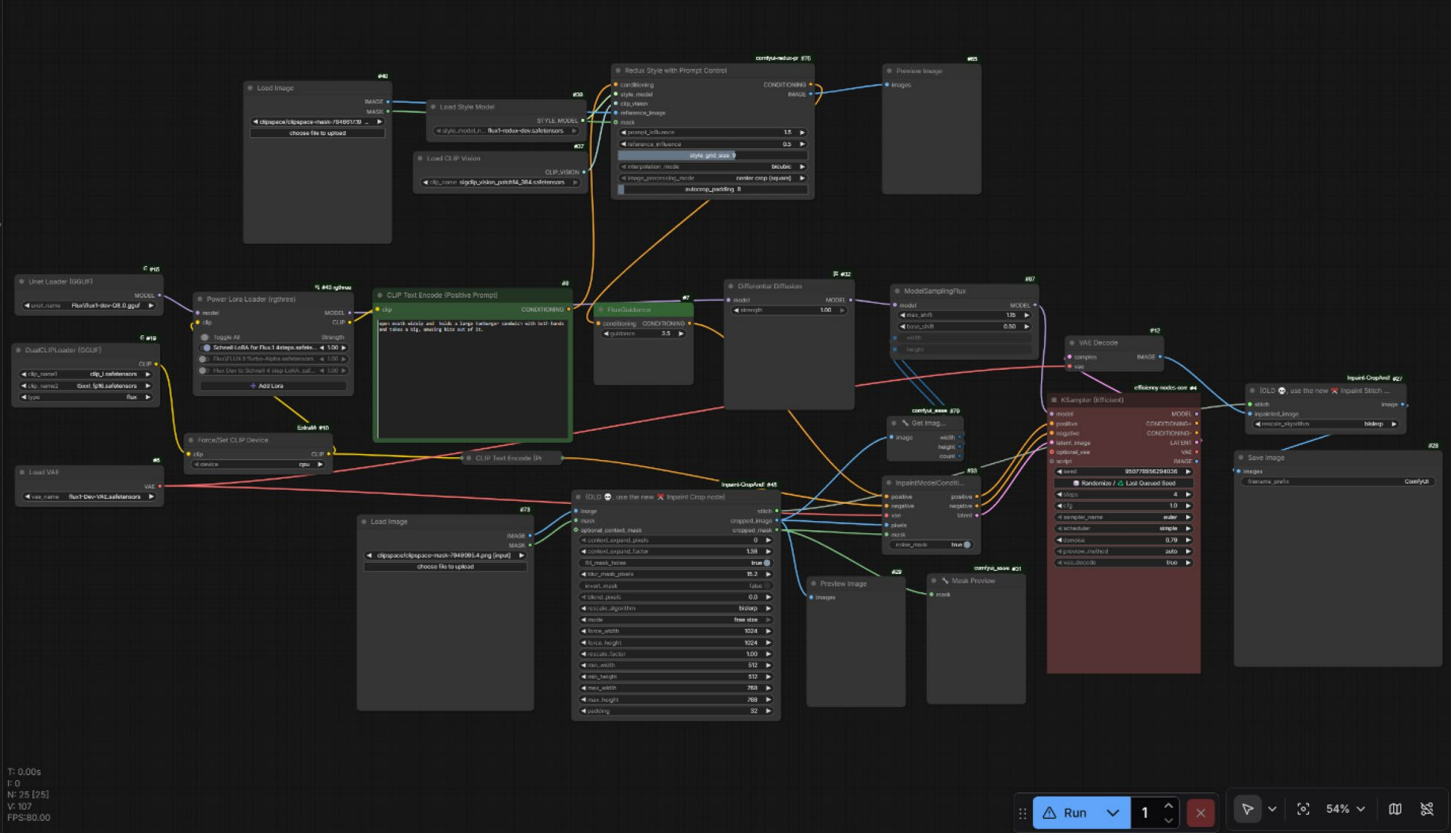
AI inpainting workflow in ComfyUI^18^.

AI-Enhanced video production AI processing was performed exclusively on still frame images extracted from source video. All video assembly occurred in DaVinci Resolve. This frame-based approach provided precise control over processed regions (background) versus preserved regions (presenter), avoiding color mismatches common with automated platforms. Figure 2 demonstrates the ComfyUI inpainting workflow.

The workflow consisted of: (1) movement analysis; (2) frame extraction at HD resolution (1920 × 1080); (3) masking creation delineating presenter regions (preserved) from background regions (replaced); (4) AI background replacement using FLUX.1-Fill (inpainting) and FLUX.1-dev (development) models^15^—two distinct models where Fill handles inpainting tasks and dev provides fine control and quality—with outpainting techniques extending spatial boundaries to create expansive studio impressions; (5) frame import as overlay in DaVinci Resolve; (6) central masking to reveal original presenter; (7) gradient feathering (50–100 pixels) for natural blending; (8) frame-by-frame review; (9) hand gesture correction via layer duplication where movements extended into AI background; (10) color matching (inherently well-matched through AI inpainting, eliminating extensive correction typical of green screen); and (11) illustration overlay (0.5–1 h per video).

**Fig. 3:**
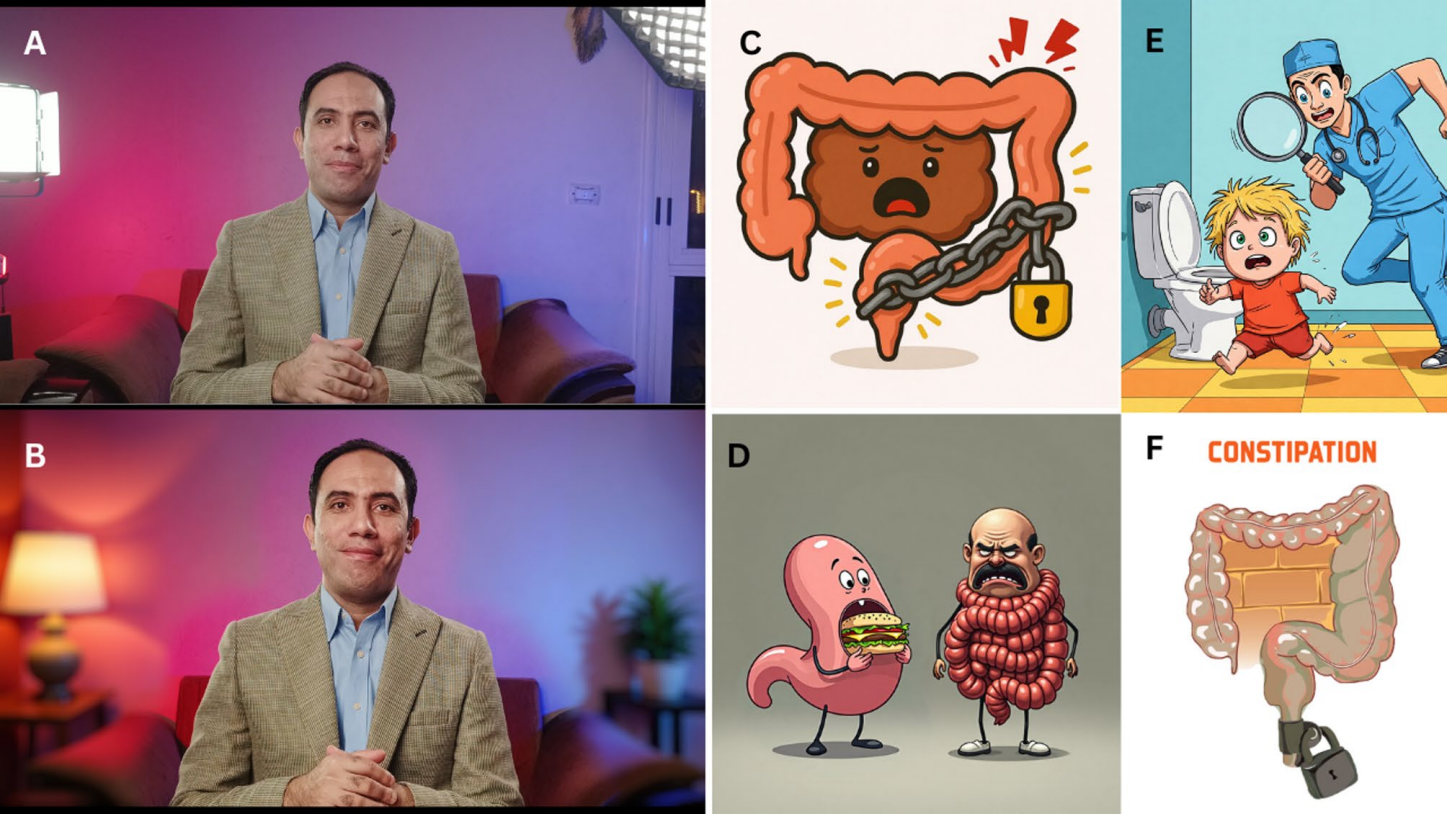
Transformation of a plain wall (**A**) into a well-decorated studio setting (**B**) achieved through AI-based inpainting. Background clutter such as windows and doors was removed, with seamless color matching and realistic integration of decorative elements and light reflections on the wall. Panels A and B feature the first author during the video capture process. Panels C and F depict two different constipation-related pathologies, each presented in simplified conceptual formats. Panel D illustrates the gastrocolic reflex. Panel E provides an additional fully AI-generated caricature showing a doctor humorously chasing the cause of constipation. Panels B, C, D, E, and F were AI-manipulated or fully created using our workflow and FLUX.1 models in ComfyUI.^18^.

AI processing was strictly limited to background enhancement. No alterations were made to presenter’s facial features, body proportions, gestures, voice, or speech content. This avoided the risk of anatomical distortions (extra fingers, distorted features) that occur in fully AI-generated videos where presenters are synthetic^16,17^. All authentic presenter features remained entirely unmodified. Figure 3 and supplementary Video 1 demonstrate the transition from plain wall recording to studio-enhanced view.

Technical implementation AI enhancement used FLUX.1-Fill and FLUX.1-dev models via ComfyUI^18^, an open-source workflow interface, with models loaded using GGUF Q8 quantization^19^reducing computational requirements to enable operation on consumer-grade laptop (Lenovo Legion 5 Pro with 6GB VRAM). GGUF Q8 quantization was selected for publication-grade quality. Inpainting used 20–30 sampling steps. Individual frame generation required 1–2 min. AI processing time ranged from 30 min to 3 h per video (iterative generation for aesthetic quality, as learner perception of professionalism was a study outcome). Post-AI processing in DaVinci Resolve required approximately 1 h. All AI-generated visual elements underwent board-certified pediatric surgeon review.

## Outcome measures

Outcome assessment was performed immediately after viewing using REDCap^20^. Primary outcomes were educational clarity (three-item, five-point Likert scale assessing presentation clarity, visual support effectiveness, and confidence in understanding) and knowledge acquisition (percentage correct on a 40-item multiple-choice examination).

The knowledge test items were mapped to predefined learning objectives for each topic and reviewed by board-certified pediatric surgeons for content relevance and clarity prior to administration. The clarity scale was designed to capture key dimensions of instructional quality (presentation clarity, visual support, and learner confidence).

Secondary outcomes included visual comfort, perceived professionalism, willingness to recommend, and perceived improvement compared with textbook-based learning.

### Statistical analysis

Continuous variables were summarized as mean ± SD. Between-group comparisons used independent-samples t-tests; categorical outcomes used chi-square tests. Effect sizes were calculated using Cohen’s d. Multivariable linear regression assessed independent associations adjusting for training level, age, prior exposure, and baseline familiarity. Significance was *p* < 0.05.

### Economic analysis

Cost comparison estimated per-video production costs including studio rental, equipment, services, hardware amortization, faculty time, and illustrations. Faculty time was valued at $50/hour for base analysis.

## Results

### Participant characteristics

Between December 2024 and September 2025, 256 trainees were screened. Sixteen (6.3%) were excluded: twelve (4.7%) had prior exposure, four (1.6%) were in fellowship. Total 240 participants were included: 120 (50%) per condition, with zero attrition. The cohort consisted predominantly of undergraduate medical students (*n* = 210, 87.5%), with smaller proportions of junior residents PGY 1–2 (*n* = 20, 8.3%) and senior residents PGY ≥ 3 (*n* = 10, 4.2%). Mean age was 23.7 years (SD 2.2, range 21–29). Baseline characteristics were well balanced (Table 1). Prior clinical exposure: 87 (72.5%) standard versus 88 (73.3%) AI-enhanced (χ²=0.02, *p* = 0.89). Baseline familiarity: mean 2.8 (SD 1.1) standard versus 2.9 (SD 1.2) AI-enhanced (t = 0.43, *p* = 0.67).

**Table 1:** Baseline characteristics (*n* = 240).

Characteristic	Standard (*n* = 120)	AI-Enhanced (*n* = 120)	*p*-value
Training level			0.94
Undergraduate students	105 (87.5%)	105 (87.5%)	
Junior residents (PGY 1–2)	10 (8.3%)	10 (8.3%)	
Senior residents (PGY ≥ 3)	5 (4.2%)	5 (4.2%)	
Age, years, mean (SD)	23.6 (2.1)	23.8 (2.3)	0.48
Male sex, n (%)	68 (56.7%)	71 (59.2%)	0.69
Prior exposure, n (%)	87 (72.5%)	88 (73.3%)	0.89
Baseline familiarity (1–5), mean (SD)	2.8 (1.1)	2.9 (1.2)	0.67

PGY=postgraduate year. Familiarity scale: 1 = no prior knowledge, 5 = very familiar.

### Primary educational outcomes

Educational clarity scores were significantly higher among AI-enhanced video participants (Table 2). Mean overall clarity was 4.52 (SD 0.46) AI-enhanced versus 3.88 (SD 0.58) standard, representing difference of 0.64 points (95% CI 0.51–0.77; t = 11.37, *p* < 0.001) and very large effect size (Cohen’s d = 1.24; 95% CI 1.00–1.48.00.48). All three scale components demonstrated superiority: presentation clarity (4.56 vs. 3.82, *p* < 0.001), visual support effectiveness (4.51 vs. 3.89, *p* < 0.001), and confidence in understanding (4.49 vs. 3.93, *p* < 0.001).

Knowledge acquisition was significantly higher in AI-enhanced group. Mean scores were 76.8% (SD 9.2) AI-enhanced versus 69.5% (SD 10.4) standard, representing difference of 7.3% points (95% CI 4.9–9.7; t = 5.93, *p* < 0.001) and large effect size (Cohen’s d = 0.76; 95% CI 0.53–0.99)—equivalent to approximately three additional correct answers per participant. Proportion achieving ≥ 80% was significantly greater in AI-enhanced group (46/120, 38.3% vs. 26/120, 21.7%; χ²=8.23, *p* = 0.004, OR = 2.25, 95% CI 1.28–3.97). Fewer AI-enhanced participants scored < 60% (7/120, 5.8% vs. 19/120, 15.8%; χ²=6.14, *p* = 0.013, OR = 0.33, 95% CI 0.13–0.82).

**Table 2:** Educational Outcomes.

Outcome	Standard (*n* = 120)	AI-enhanced (*n* = 120)	Difference (95% CI)	*p*-value	Effect size (95% CI)
Primary outcomes					
Clarity score (1–5)	3.88 ± 0.58	4.52 ± 0.46	0.64 (0.51–0.77)	< 0.001	d = 1.24 (1.00–1.48.00.48)
Knowledge score (%)	69.5 ± 10.4	76.8 ± 9.2	7.3 (4.9–9.7)	< 0.001	d = 0.76 (0.53–0.99)
Secondary outcomes					
Visual comfort (1–5)	3.91 ± 0.60	4.48 ± 0.47	0.57 (0.43–0.71)	< 0.001	d = 1.07 (0.83–1.31)
Professionalism (1–5)	3.89 ± 0.62	4.51 ± 0.48	0.62 (0.48–0.76)	< 0.001	d = 1.13 (0.88–1.37)
Would recommend, n (%)	76 (63.3%)	105 (87.5%)	-	< 0.001	OR = 4.02 (2.17–7.44)
Improved vs. reading, n (%)	71 (59.2%)	101 (84.2%)	-	< 0.001	OR = 3.68 (2.03–6.67)

Values are mean ± SD or n (%). d=Cohen’s d; OR=odds ratio.

### Secondary outcomes and subgroup analyses

Secondary outcomes consistently favored AI-enhanced videos. Participants reported greater visual comfort (mean 4.48 vs. 3.91; t = 8.16, *p* < 0.001; d = 1.07) and professionalism (mean 4.51 vs. 3.89; t = 8.62, *p* < 0.001; d = 1.13). They were significantly more likely to recommend AI-enhanced format (105/120, 87.5% vs. 76/120, 63.3%; χ²=18.82, *p* < 0.001, OR = 4.02) and perceive it as superior to textbook learning (101/120, 84.2% vs. 71/120, 59.2%; χ²=18.22, *p* < 0.001, OR = 3.68).

Knowledge improvements were observed across all five topics. Mean differences consistently favored AI-enhanced condition with significant gains: functional constipation (7.6% points, *p* < 0.001, d = 0.71), undescended testes (7.3% points, *p* < 0.001, d = 0.72), bowel management (5.4% points, *p* = 0.001, d = 0.68), hypospadias (8.7% points, *p* < 0.001, d = 0.84), and ureteropelvic junction obstruction (7.4% points, *p* < 0.001, d = 0.78). No single topic disproportionately drove overall effect.

Educational benefits were consistent across training levels. Among undergraduate students (*n* = 210), AI-enhanced videos showed higher clarity (difference 0.66 points, 95% CI 0.53–0.79; *p* < 0.001; d = 1.27) and knowledge (difference 7.6% points, 95% CI 5.1–10.1; *p* < 0.001; d = 0.79). Among junior residents (*n* = 20), AI-enhanced videos showed higher clarity (difference 0.52 points, 95% CI 0.01–1.03; *p* = 0.048; d = 0.99) and knowledge (difference 5.7% points, *p* = 0.028; d = 0.56), though power was limited.

After adjustment for training level, age, prior exposure, and baseline familiarity, AI-enhanced videos remained independently associated with higher clarity (adjusted estimate 0.62 points, 95% CI 0.49–0.75, *p* < 0.001) and knowledge (7.1% points, 95% CI 4.7–9.5, *p* < 0.001). No significant interaction between video condition and training level was observed for clarity (F = 0.84, *p* = 0.43) or knowledge (F = 0.65, *p* = 0.52).

### Economic analysis

Cost comparison demonstrated substantial economic advantage for AI-enhanced production (Table 3). Professional studio production typically requires studio rental ($500-2,000/day), professional equipment ($1,000–5,000/session), and videographer/editing services ($500-1,500/video), totaling $2,000–8,500 per video. AI-enhanced approach required one-time hardware investment (~$1,200, amortized to $21–33 per video over three years assuming 36 videos) and faculty time. Total active faculty time was 7–10 h per video at $50/hour ($350–500). Total per-video cost was $371–533 without illustrations or $396–583 with illustrations, representing 72–94% cost reduction. Break-even was reached after 1–2 videos. For five-video series, total savings ranged from $8,000 to $40,000. Cost advantage persists even valuing faculty time at institutional rates ($100–200/hour), as total AI-enhanced costs ($700-2,000/video) remain below professional studio ($2,000–8,500/video).

**Table 3:** Economic Comparison.

Component	Traditional studio	Local AI-enhanced
Studio rental	$500-2,000	$0
Professional equipment	$1,000–5,000	$0
Videographer/editing services	$500-1,500	$0
Hardware (amortized)	N/A	$21–33
Faculty time:		
Content development	2–3 h ($100–150)*	2–3 h ($100–150)
Recording	1 h ($50)*	1 h ($50)
Basic editing (silences/cuts)**	1–2 h ($50–100)***	1–2 h ($50–100)
AI frame extraction/masking	N/A	20 min ($17)
AI processing oversight	N/A	1 h ($50)
DaVinci post-production	N/A	1 h ($50)
Illustration overlay (optional)	0.5–1 h ($25–50)	0.5–1 h ($25–50)
Expert validation	1 h ($50)	1 h ($50)
Total per video	**$2**,150-8,**750**	**$371–583**
Cost reduction	**-**	**72–94%**

*Minimal faculty oversight time as professional services handle production **Applies to both methods ***Professional studio may include this in videographer services.

## Discussion

This study demonstrates that locally deployed AI-enhanced educational videos significantly improve perceived educational clarity and objective knowledge acquisition in pediatric surgical teaching compared with standard faculty-recorded videos, with consistent improvements across learner levels and topics, while substantially reducing costs. The approach eliminates green screen infrastructure and its technical challenges.

The improvement observed addresses a critical gap. Pediatric surgery exposure remains severely limited, with residents completing only 33.8 designated cases (3.7% of volume), down from 47.7 cases, with ACGME requiring just 20 cases^1,2^. In many medical schools, pediatric surgery is taught within general surgery curriculum for undergraduates, making video-based resources essential. Video-based education has become essential as Education 4.0 emphasizes technology-enhanced, student-centered learning.

The knowledge improvement magnitude (7.3% points, Cohen’s d = 0.76) compares favorably with prior meta-analyses showing large effects in dentistry (d = 2.18) and moderate effects in medicine (d = 0.67)^9^. Studies demonstrate significant normalized learning gains from video-based education, with interactive videos achieving 49–70% gains depending on format^8^. Our results suggest AI enhancement provides additive value beyond standard recording. The very large effect size for perceived clarity (d = 1.24) likely reflects isolation of visual presentation as the sole manipulated variable, with identical content across groups.

This aligns with Cognitive Theory of Multimedia Learning, which posits that multimedia learning engages separate channels for processing words and pictures, with limited working memory requiring appropriate cognitive processing^10^. Three important goals are reducing extraneous processing, managing essential processing, and fostering generative processing. Enhanced visual presentation may improve engagement without altering content delivery, consistent with student-centered principles emphasizing learner autonomy and technology integration. Dual coding theory suggests content presented through verbal and visual channels improves information processing. An AI Educational Video Assistant designed using these principles received positive evaluations across engagement, organization, clarity, and usability^21^.

Findings are particularly relevant for programs lacking professional media resources. Locally deployed, open-source AI tools offer pragmatic solution preserving faculty autonomy, avoiding recurring subscriptions, and maintaining institutional control. The 72–94% cost reduction has particular relevance for resource-limited settings where traditional infrastructure is unavailable. The most expensive component of video-based education modules is time cost, which is highly variable depending on the level of clinical seniority of the individuals involved^22^.

This aligns with Education 4.0 goals. Practical solutions include recording sessions onto DVDs, reaching over 140 surgical trainees across four countries^23^. The UN Global Surgery Learning Hub provides e-learning courses to over 11,000 learners from 190 countries, with 55% from low- or lower-middle income countries^24^. Our approach complements these efforts by enabling local production without studios or green screen infrastructure.

Professional-quality enhancement was achieved using consumer-grade hardware through GGUF quantization techniques^19^. GGUF Q8 quantization reduced computational requirements, enabling operation on standard consumer laptops with modest VRAM (6GB), making this technology particularly accessible for pediatric surgery education in low- and middle-income countries. Our frame-based workflow, in which AI processing was restricted to background regions, preserved presenter authenticity while avoiding the technical limitations of green screen recording. This approach reduced lighting and color-matching constraints and enabled visually consistent backgrounds without specialized studio infrastructure.

Additional innovations included outpainting to extend spatial boundaries, creating expansive studios from confined spaces; HD-resolution AI processing with 4 K output; elimination of green screen infrastructure and replacement costs; and AI audio enhancement using free tools. These collectively reduce barriers to high-quality production. Commercially available technology allows filming high-quality content at minimal cost using smartphone cameras^25^. Our method bridges this gap by combining low-cost recording with AI-based enhancement.

The American College of Surgeons multidisciplinary consensus establishes that AI methods and AI-enabled metrics should have strong evidence of validity to facilitate adoption by surgeons (90% agreement), and that benchmarking against expert assessments is essential for skill assessment (90% agreement), though this is susceptible to biases inherent in subjective human opinions (85% agreement)^26^. Only 44% of published AI models in pediatric surgery were interpretable, just 6% were both interpretable and externally validated, and 40% had high risk of bias^27^. Our approach limits AI processing to background replacement while preserving all authentic presenter features through selective masking, with board-certified pediatric surgeon validation.

In this study, AI functioned strictly as a visual enhancement tool rather than a content generator. By restricting processing to background elements, anatomical accuracy and presenter authenticity were preserved, with all materials reviewed by board-certified pediatric surgeons. This controlled use of AI aligns with current consensus recommendations for responsible adoption in surgical education.

From practical implementation perspective, AI-enhanced production required more faculty time (7–10 vs. 2–4 h) but eliminated professional service costs ($2,000–8,500) and green screen infrastructure. The high cost of producing good-quality video-films for teaching has historically hindered the use of this method^28^. This trade-off favors AI enhancement when faculty time is institutionally subsidized, making the approach attractive for academic centers where faculty contributions are valued but external budgets are limited. Even valuing faculty time at institutional rates ($100–200/hour), cost advantages persist.

Key cost ingredients for video-based education include faculty time (median >$170 per physician), equipment (median >$170), and learner time (median $525 per physician)^29^. Programs prioritizing efficiency could reduce time by accepting functional rather than refined backgrounds. The primary barrier to effective AI integration is not technological sophistication, but critical infrastructure deficiencies including institutional implementation structures, sustainable funding, and rigorous research methodologies^30^. Our approach addresses technological and cost barriers while acknowledging that successful implementation requires institutional support structures.

The transition from language models to multimodal visual AI represents an important paradigm shift^12^. Multimodal AI utilizing contrastive language-image pretraining (CLIP) enables sophisticated manipulation of educational media previously requiring professional studios. Generative AI can transform educational content into multimodal formats, potentially improving comprehension through alignment with Cognitive Theory of Multimedia Learning^13^. The FLUX.1-Fill and FLUX.1-dev models represent state-of-the-art inpainting technology, with Fill handling inpainting tasks and dev providing fine control and quality optimization. This enables precise background replacement while preserving presenter authenticity, critical requirements that would be compromised by fully automated approaches^15^.

AI holds significant promise for personalizing health professional education, with an increasing trend in AI applications, particularly in surgical education^31^. AI’s affordances—expressivity, interactivity, multimodality—map to educational experiences such as interactive tutoring, though meaningful use demands critical awareness of AI’s operational limits, ethical boundaries, and technical mechanisms.

Although video-based education and AI-assisted learning have each demonstrated educational value^7^, the specific use of locally deployed AI for visual enhancement of pediatric surgery lecture videos has not previously been evaluated. The present findings therefore provide preliminary evidence for this application.

The greatest promise lies in hybrid models where AI augments rather than replaces faculty mentorship. AI has the potential to be a powerful adjunct to surgical education if deployed thoughtfully and ethically, complementing rather than replacing the vital educator-learner relationship^32^. AI cannot replace the critical role of educators and must be used as complementary tool with robust governance frameworks.

### Limitations

This study has several limitations. First, the quasi-experimental, non-randomized, post-test-only design introduces potential selection bias and limits causal inference, although baseline characteristics were well balanced and multivariable adjustment was performed. Second, outcomes were assessed immediately after video viewing; therefore, long-term knowledge retention and transfer to clinical performance were not evaluated. Third, although the knowledge assessment instrument was mapped to learning objectives and reviewed by faculty, formal psychometric validation was not performed. This may limit interpretation of absolute score levels, although relative between-group differences remain informative. Fourth, the observed improvements in perceived clarity may partially reflect enhanced production quality and novelty effects rather than instructional superiority alone. Fifth, the study was conducted at a single institution with a predominance of undergraduate learners, which may limit generalizability to other training environments or learner populations. Finally, the intervention evaluated lecture-based educational videos and does not address operative or procedural video instruction, where different educational dynamics may apply.

## Conclusions

Locally deployed AI-enhanced educational videos were associated with significantly improved perceived clarity and short-term knowledge acquisition compared with standard faculty-recorded videos, while achieving substantial reductions in production costs. By restricting AI use exclusively to environmental enhancement and preserving all authentic presenter features, this approach addresses key concerns regarding anatomical accuracy and educational integrity. These findings suggest that practical, open-source AI tools can enhance the accessibility and professionalism of pediatric surgery educational content, particularly in resource-limited settings. Further multicenter, randomized studies with longitudinal follow-up are needed to assess knowledge retention, clinical impact, and broader generalizability across surgical training contexts.

## Supplementary Information

Below is the link to the electronic supplementary material.

Supplementary Material 1

## Data Availability

The datasets generated and analyzed during the current study are available from the corresponding author upon reasonable request.
