# Supplementary material for: Educational impact and cost efficiency of AI-enhanced videos in pediatric surgery training: a quasi-experimental study
Source: Sci Rep. 2026 Mar 12;16:8952. doi: 10.1038/s41598-026-39961-y (PMC12987918; doi:10.1038/s41598-026-39961-y)
Supplement: Supplementary file 1 — Supplementary Material 1 [file 41598_2026_39961_MOESM1_ESM.docx]

1. **TREND Checklist for Reporting Quasi-Experimental Educational Studies**

| **Item No.** | **Item** | **Location in Manuscript** |
| --- | --- | --- |
| **Title and Abstract** |  |  |
| 1 | Information on intervention, study design, methods, and primary outcomes | Abstract |
| **Introduction** |  |  |
| 2 | Scientific background and rationale | Introduction, paragraphs 1-4 |
| 3 | Specific objectives and hypotheses | Introduction, final paragraph |
| **Methods** |  |  |
| 4 | Study design (quasi-experimental, post-test only) | Methods, Study Design section |
| 5 | Eligibility criteria for participants | Methods, Study Design section |
| 6 | Settings and locations where data collected | Methods, Study Design section |
| 7 | Interventions: AI-enhanced vs standard videos | Methods, Video Production section |
| 8 | Clearly defined primary and secondary outcomes | Methods, Outcome Measures section |
| 9 | Sample size determination | Methods, Study Design section |
| 10 | Method of assignment to conditions | Methods, Study Design section |
| 11 | Blinding (outcome assessors) | Methods, Outcome Measures section |
| 12 | Statistical methods | Methods, Statistical Analysis section |
| **Results** |  |  |
| 13 | Flow of participants (screening, enrollment, allocation, analysis) | Results, Figure 4; Participant Characteristics |
| 14 | Baseline demographic and clinical characteristics | Results, Table 1 |
| 15 | Numbers analyzed for each outcome | Results, Tables 2-3 |
| 16 | Outcomes and estimation with confidence intervals | Results, Tables 2-3; Primary/Secondary Outcomes |
| 17 | Subgroup analyses | Results, Secondary Outcomes section |
| 18 | Adverse events | Not applicable (educational study) |
| **Discussion** |  |  |
| 19 | Interpretation of results consistent with study design | Discussion, paragraphs 1-6 |
| 20 | Generalizability (external validity) | Discussion, Limitations section |
| 21 | Overall evidence | Discussion, all paragraphs |
| **Other** |  |  |
| 22 | Funding sources and role | Not included (no external funding) |

1. **GREET Checklist (Educational Interventions)**

| **Domain** | **Item** | **Reported** |
| --- | --- | --- |
| **Setting** | Educational context described | ✓ (Methods) |
| **Learning Outcomes** | Primary outcomes clearly defined | ✓ (Methods) |
| **Curriculum Integration** | How intervention fits into curriculum | ✓ (Introduction, Methods) |
| **Intervention Description** | Detailed description of educational intervention | ✓ (Methods, Video Production) |
| **Comparison** | Control condition described | ✓ (Methods) |
| **Duration** | Duration of exposure/intervention | ✓ (Methods: 6-10 min videos) |
| **Fidelity** | Implementation consistency ensured | ✓ (Methods) |
| **Assessment** | Valid assessment tools used | ✓ (Methods, Cronbach's α=0.86) |
| **Data Analysis** | Appropriate statistical methods | ✓ (Methods, Results) |
| **Results** | Complete outcome data reported | ✓ (Results, Tables 2-3) |
